# Physician-led telemedical care enhances blood pressure control in hypertension: a randomized-controlled pilot study (REMOTE-control-HTN)

**DOI:** 10.1093/ehjopen/oeag061

**Published:** 2026-04-21

**Authors:** Paula Sagmeister, Natalie Fischer, Diana Eckert, Luise Mentzel, Parham Shahidi, Charlotte Wolff, Holger Thiele, Karl Fengler

**Affiliations:** Department of Cardiology, Heart Centre Leipzig at Leipzig University and Leipzig Heart Science, Struempellstr. 39, Leipzig 04289, Germany; Department of Cardiology, Heart Centre Leipzig at Leipzig University and Leipzig Heart Science, Struempellstr. 39, Leipzig 04289, Germany; Department of Cardiology, Heart Centre Leipzig at Leipzig University and Leipzig Heart Science, Struempellstr. 39, Leipzig 04289, Germany; Department of Cardiology, Heart Centre Leipzig at Leipzig University and Leipzig Heart Science, Struempellstr. 39, Leipzig 04289, Germany; Department of Cardiology, Heart Centre Leipzig at Leipzig University and Leipzig Heart Science, Struempellstr. 39, Leipzig 04289, Germany; Department of Cardiology, Heart Centre Leipzig at Leipzig University and Leipzig Heart Science, Struempellstr. 39, Leipzig 04289, Germany; Department of Cardiology, Heart Centre Leipzig at Leipzig University and Leipzig Heart Science, Struempellstr. 39, Leipzig 04289, Germany; Department of Cardiology, Heart Centre Leipzig at Leipzig University and Leipzig Heart Science, Struempellstr. 39, Leipzig 04289, Germany

## Abstract

**Aims:**

Despite advances in antihypertensive therapy, blood pressure (BP) control remains inadequate in many patients due to limited follow-up and insufficient medication titration.

**Methods and results:**

This single-centre, randomized controlled pilot trial was conducted at a tertiary academic hospital in Germany between December 2023 to September 2024. Sixty adults with uncontrolled hypertension (office BP >140/90 mmHg despite antihypertensive medication) were randomized 1:1 to standard care or physician-led telemedical care. All participants performed home BP measurements twice daily using validated telemedical devices. The intervention group received structured biweekly calls for BP review and medication optimization. The control group continued care with their general practitioners. The primary endpoint was time in target range (TTR) over 6 months, defined as the percentage of home BP readings below guideline thresholds (<130/80 mmHg for age <65, <140/90 mmHg for age ≥65). Group comparisons used *t*-tests. The study was registered at CinlicalTrials.gov Identifier [NCT07049289]. Fifty-six patients completed 6-month follow-up (mean age 61 ± 13 years, 61% male). Baseline BP was 161 ± 17/97 ± 12 mmHg. At 6 months, systolic BP decreased by −15.0 ± 9.8 mmHg in the intervention group vs. −4.0 ± 8.7 mmHg in the control group (*P* < 0.001). Mean systolic TTR was significantly higher in the telemedical care group (52.2 ± 24.2% vs. 36.0 ± 29.2%, *P* = 0.028), as was diastolic TTR (59.0 ± 31.0% vs. 37.1 ± 34.4%, *P* = 0.016), and total TTR (42.9 ± 27.5% vs. 24.8 ± 29.7%, *P* = 0.021). Measurement adherence was superior in the intervention group (83.2 ± 15.4% vs. 70.5 ± 25.9%, *P* = 0.033).

**Conclusion:**

Physician-supported telemedical care significantly improved BP control and measurement adherence over 6 months. Larger trials are needed to assess long-term clinical outcomes.

## Graphical Abstract

**Graphical Abstract:**
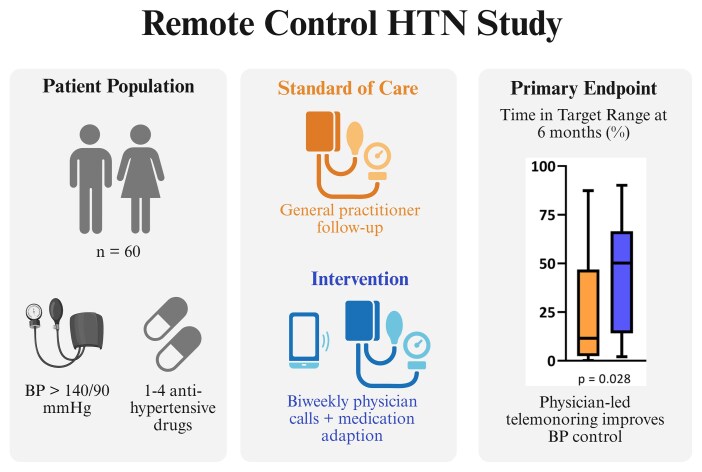

## Introduction

Arterial hypertension is one of the most prevalent chronic diseases, affecting over 1.2 billion people worldwide, and remains the leading modifiable risk factor for cardiovascular morbidity and mortality.^1,2^

Despite effective medications and lifestyle interventions, fewer than 50% of patients achieve recommended blood pressure (BP) targets.^3,4^ The 2024 European Society of Cardiology (ESC) guidelines now recommend a BP target < 130/80 mmHg for all patients if tolerated,^5^ further widening the gap between guideline targets and real-world BP control.

Effective hypertension management requires regular BP monitoring, sustained lifestyle changes, adherence to therapy, and timely medication adjustments^6,7^—challenges in routine care due to resource constraints and limited access.

Telemonitoring has emerged as a promising tool to address these challenges.^8^ In heart failure, it has been shown to reduce mortality and hospitalizations,^9^ leading to reimbursement by statutory health insurance in several countries. Evidence for telemedical care in the management of hypertension is less conclusive. While some smaller trials have reported positive effects of telemedical interventions in patients with elevated BP or prehypertension,^10–12^ large-scale evidence on the effectiveness of telemonitoring in routine care for patients with manifest hypertension is limited. Therefore, this study aimed to investigate whether telemonitoring, combined with regular telephone consultations, can improve BP control in patients with uncontrolled arterial hypertension in a real-world outpatient setting.

## Methods

REMOTE-Control-HTN is a prospective, randomized, controlled, single-centre, pilot trial conducted at a tertiary care centre in Germany. It was designed to evaluate the effectiveness of telemedical care in patients with uncontrolled hypertension. The study was approved by the local ethics committee, conducted in accordance with the Declaration of Helsinki, and registered at ClinicalTrials.gov: NCT07049289.

### Study participants

Adults (≥18 years) with uncontrolled hypertension (office BP >140/90 mmHg) despite ongoing treatment with 1–4 antihypertensive drug classes were eligible. Exclusion criteria included pregnancy, participation in other randomized trials, or legal supervision or guardianship. All participants provided written informed consent prior to enrollment.

### Randomization and intervention

Participants were randomized 1:1 to telemedical or standard care for the 6-month intervention phase. Randomization was performed using a computer-based algorithm without stratification.

All patients received a validated and regularly calibrated, upper-arm, semi-automatic, oscillometric home BP monitoring device with automated data transfer via the GSM mobile network (Tel-O-Graph GSM, IEM, Germany). The device has undergone formal validation according to the British Hypertension Society protocol^13^ and is listed as recommended by STRIDE BP and Medaval. Patients were instructed on correct BP measurement technique according to the current ESC recommendations,^5^ including resting in a seated position, appropriate cuff size, placement at heart level, and avoidance of caffeine or physical activity immediately before measurement. At enrollment, mid-upper-arm circumference was measured and cuff-size was selected accordingly (S 20–24 cm; M 24–32 cm, L 32–38 cm, XL 38–55 cm). At home, patients were instructed to measure twice daily, in the morning and evening, with two consecutive measurements. Each measurement was time-stamped and transmitted automatically to a secure web-based portal accessible to the study team. A maximum of four readings per day were analysed. If more than four readings were transmitted on any given day, the highest and lowest values were retained for analysis to limit overrepresentation of days with repeated measurements.

Participants in the telemedical care group were contacted by a physician from the study team via telephone twice per month. During these structured calls BP values, medication intake, treatment adherence, adverse effects, and lifestyle factors were reviewed. Treatment recommendations, including dose titrations, medication changes, prompt initiation of alternative agents in case of intolerance, or reinforcement of lifestyle measures, were discussed and implemented in collaboration with the patient. Lifestyle counselling included reinforcement of guideline-based measures, such as salt restriction, healthy diet, weight management, regular physical activity, moderation of alcohol, smoking cessation, and sleep hygiene. ‘Healthy diet’ was discussed in general terms, but no standardized dietary programme was prescribed. Lifestyle adherence was not systematically monitored or quantified during follow-up. Prescriptions were either mailed to patients or transmitted electronically on the patient’s health card, as customary in Germany. In patients with normotensive BP, the frequency of telemedical contact was reduced to once every 4 weeks.

The control group received standard hypertension care, with medication adjustments performed by the general practitioner or, if already under follow-up or clinically indicated, by our outpatient hypertension clinic. There was no additional input from the study team, unless technical problems occurred, failure to transmit data for more than 3 consecutive days, or elevated BP values >200/120 mmHg. In these cases, the study team called the patients to instruct them to continue BP monitoring or promptly consult their general practitioner for medication adjustment.

### Blood pressure targets

Individual BP targets were set according to current ESC guidelines: <130/80 mmHg for patients <65 years, and <140/90 mmHg for ≥65 years.^14^ For patients <65 years who experienced symptoms under lower BP levels despite optimization efforts over a period of at least 4 weeks, the target range could be adjusted to <140/90 mmHg. In patients with a formal target range of <130/80 mmHg where further systolic BP reduction was deemed clinically unfeasible due to low systolic values and a diastolic BP >80 mmHg, the target range was adjusted to <130/90 mmHg. All modifications were discussed in the steering committee, documented accordingly, and considered during analysis.

### Adherence and safety monitoring

BP measurement adherence was continuously monitored via automated device transmission and checked at least biweekly by the study team. Adherence to medication was assessed through laboratory-based detection of antihypertensive agents in blood and urine samples at baseline and after 6 months. In addition to the medication adherence testing, the follow-up visit at 6 months included medication reviews and clinical evaluations.

Safety analyses were performed on all 60 randomized patients, including those who withdrew before completing the 6-month follow-up, to comprehensively capture all adverse events occurring during the first 6 months. Adverse events were documented continuously in the intervention group during telephone contacts, and in the control group at the 6-month visit.

### Outcomes

The primary end-point was time in BP target range (TTR) from randomization through 6 months, defined as the proportion of all valid home BP measurements falling below the individualized treatment target.

### Statistical analysis

Descriptive statistics were used to present patient characteristics, BP outcome measures, adherence to home BP monitoring and side effects. They were shown as mean ± standard deviation (SD) for normally distributed variables, and median with interquartile range (IQR) for non-normally distributed variables. Between-group comparisons were performed using independent-samples *t*-tests for continuous variables, and χ^2^-test or Fisher’s exact tests for categorical variables, as appropriate.

The primary end-point—TTR over 6 months—was analysed in a modified intention-to-treat population including all randomized participants who did not withdraw consent for use of their data and with available home BP transmissions (*n* = 56). Safety analyses were performed in all randomized patients (*n* = 60). For the primary analysis, total TTR (both systolic and diastolic BP values were simultaneously within target), systolic and diastolic TTR were calculated.

To analyse longitudinal BP trajectories over 26 weeks, a repeated-measures ANOVA was performed, including time × group interaction term to assess differential changes between interaction and control group. Given that the sphericity assumption tested by Mauchly’s test was violated, Greenhouse–Geisser correction was applied for all further analyses.

Subgroup analyses were performed to assess differences in TTR across predefined clinical categories, including age, sex, body mass index (BMI), baseline systolic BP, BP target, number of antihypertensive drugs at baseline, and measurement adherence. For each subgroup, the difference in systolic TTR between intervention and control groups was calculated, with corresponding 95% confidence intervals (CI) and *P*-values. Additionally, *P*-values for interaction were calculated in order to assess whether the intervention effect differed across subgroups. Forest plots were generated to illustrate subgroup effects.

Statistical calculations were performed using SPSS version 28.0.0 (IBM). Figures were created using Graph Pad Prism version 8.0.1 (GraphPad Software, San Diego, CA, USA) or PowerPoint 2016 (Microsoft, Redmond, WA, USA).

## Results

### Patient enrollment and follow-up

**Figure 1:**
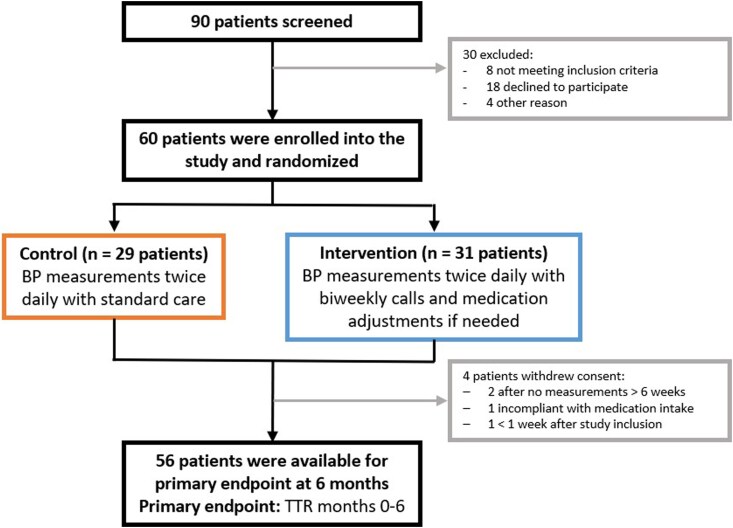
Study flow. Flowchart of patient screening, inclusion, and drop-out at 6 months. A total of 90 patients were screened, of whom 60 were enrolled and randomized 1:1 to either telemedical care (*n* = 31) or standard care (*n* = 29). Four patients in the intervention group withdrew consent prior to the 6-month primary end-point assessment, leaving 56 patients for analysis. Abbreviations: BP, blood pressure; TTR, time in target range.

A total of 60 patients with uncontrolled arterial hypertension were enrolled between December 2023 and September 2024 (*Figure 1*). Of these, 31 patients were randomized to the telemedical care group and 29 to the standard care group.

Four patients ultimately withdrew consent and were excluded from the analysis. The reasons for withdrawal included prolonged absence of BP measurements and failure to contact (*n* = 2), non-adherence to antihypertensive medication (*n* = 1), and early withdrawal of consent within the first month (*n* = 1).

### Baseline characteristics

Baseline characteristics and antihypertensive medication are shown in *Table 1*. The overall mean age was 62 ± 13 years, with 60% of patients being male and predominantly of Caucasian ethnicity (98%). The average BMI was 29.3 ± 4.6 kg/m^2^.

**Table 1:** Baseline characteristics and medication

		All patients (*n* = 60)	Control (*n* = 29)	Intervention (*n* = 31)	*P*-value^a^
**Age, years**		62 ± 13	62 ± 14	61 ± 13	0.876
**Sex (male), *n* (%)**		36 (60.0)	18 (62.1)	18 (58.1)	0.752
**Race, *n* (%)**	**White**	59 (98.3)	28 (96.6)	31 (100)	0.297
	**Other**	1 (1.7)	1 (3.4)	0 (0)	
**Body mass index, kg/m^2^**		29.3 ± 4.6	28.6 ± 4.4	30.0 ± 4.8	0.259
**Office systolic BP mmHg**		162 ± 17	160 ± 11	164 ± 22	0.394
**Office diastolic BP, mmHg**		98 ± 13	95 ± 11	100 ± 14	0.164
**Heart rate, bpm**		69 ± 9	69 ± 9	70 ± 10	0.677
**Smoking, *n* (%)**	**No**	35 (58.3)	13 (44.8)	22 (71.0)	0.022
	**Currently**	13 (21.7)	6 (20.7)	7 (22.6)	
	**Previously**	12 (20.0)	10 (34.5)	2 (6.5)	
**Diabetes mellitus, *n* (%)**		3 (5.0)	2 (6.9)	1 (3.2)	0.606
**Peripheral artery disease, *n* (%)**		2 (3.3)	2 (6.9)	0 (0)	0.229
**Coronary artery disease, *n* (%)**		8 (13.3)	4 (13.8)	4 (12.9)	>0.999
**Previous stroke, *n* (%)**		1 (1.7)	0 (0)	1 (3.2)	>0.999
**Heart failure, *n* (%)**		4 (6.7)	4 (13.8)	0 (0)	0.049
**Atrial fibrillation, *n* (%)**		2 (3.3)	1 (3.4)	1 (3.2)	>0.999
**Dyslipidaemia, *n* (%)**		38 (63.3)	15 (51.7)	23 (74.2)	0.071
**Serum creatinine, µmol/L**		83 ± 17	83 ± 17	82 ± 18	0.861
**eGFR, mL/min/1.73 m^2^**		80 ± 17	80 ± 18	80 ± 17	0.967
**Number of antihypertensive drug classes**		2.4 ± 1.0	2.4 ± 0.9	2.3 ± 1.1	0.461
**Angiotensin-converting enzyme inhibitors, *n* (%)**		20 (33.3)	9 (31.0)	11 (35.5)	0.715
**Angiotensin receptor antagonists, *n* (%)**		37 (61.7)	18 (62.1)	19 (61.3)	0.951
**Renin antagonists, *n* (%)**		1 (1.7)	1 (3.4)	0 (0)	NA
**Beta blockers, *n* (%)**		31 (51.7)	16 (55.2)	15 (48.4)	0.599
**Calcium channel blockers, *n* (%)**		24 (40.0)	11 (37.9)	13 (41.9)	0.752
**Diuretics 1, *n* (%)**		20 (33.3)	13 (44.8)	7 (22.6)	0.068
**Diuretics 2, *n* (%)**		1 (1.7)	0 (0)	1 (3.2)	>0.999
**Aldosteron antagonist, *n* (%)**		1 (1.7)	1 (3.4)	0 (0)	0.483
**Vasodilators, *n* (%)**		1 (1.7)	0 (0)	1 (3.2)	>0.999
**Alpha blockers, *n* (%)**		3 (5.0)	1 (3.4)	2 (6.5)	>0.999
**Sympatholytics, *n* (%)**		3 (5.0)	2 (6.9)	1 (3.2)	0.605

^a^Continuous variables were compared using independent-samples *t*-tests, while categorical variables were compared using χ^2^ tests or Fisher’s exact tests when any subgroup had an expected count <5. Statistical significance was defined as *P* < 0.05.

Abbreviations: BP, blood pressure.

At baseline, mean office systolic and diastolic BPs were 162 ± 17 mmHg and 98 ± 13 mmHg, respectively. An initial target BP of <130/80 mmHg was set for 60% of participants, while 40% aimed for <140/90 mmHg. In four patients, BP targets were adjusted during follow-up, as described in the methods section.

Common comorbidities included dyslipidaemia (63%), history of smoking (42%), coronary artery disease (13%), and diabetes (5%). Renal function was preserved across the cohort, with a mean estimated glomerular filtration rate of 80 ± 17 mL/min/1.73 m^2^. Baseline characteristics were generally evenly balanced between the two groups, with the exception of a higher prevalence of smoking history in the control group (35% vs. 7%, *P* = 0.022).

Participants were treated with an average of 2.4 ± 1.0 antihypertensive drug classes at baseline (*Table 1*). The most frequently prescribed classes were angiotensin receptor antagonists (62%), beta-blockers (52%), calcium channel blockers (49%), angiotensin-converting enzyme inhibitors (33%), and diuretics (33%).

### Blood pressure outcomes

**Figure 2:**
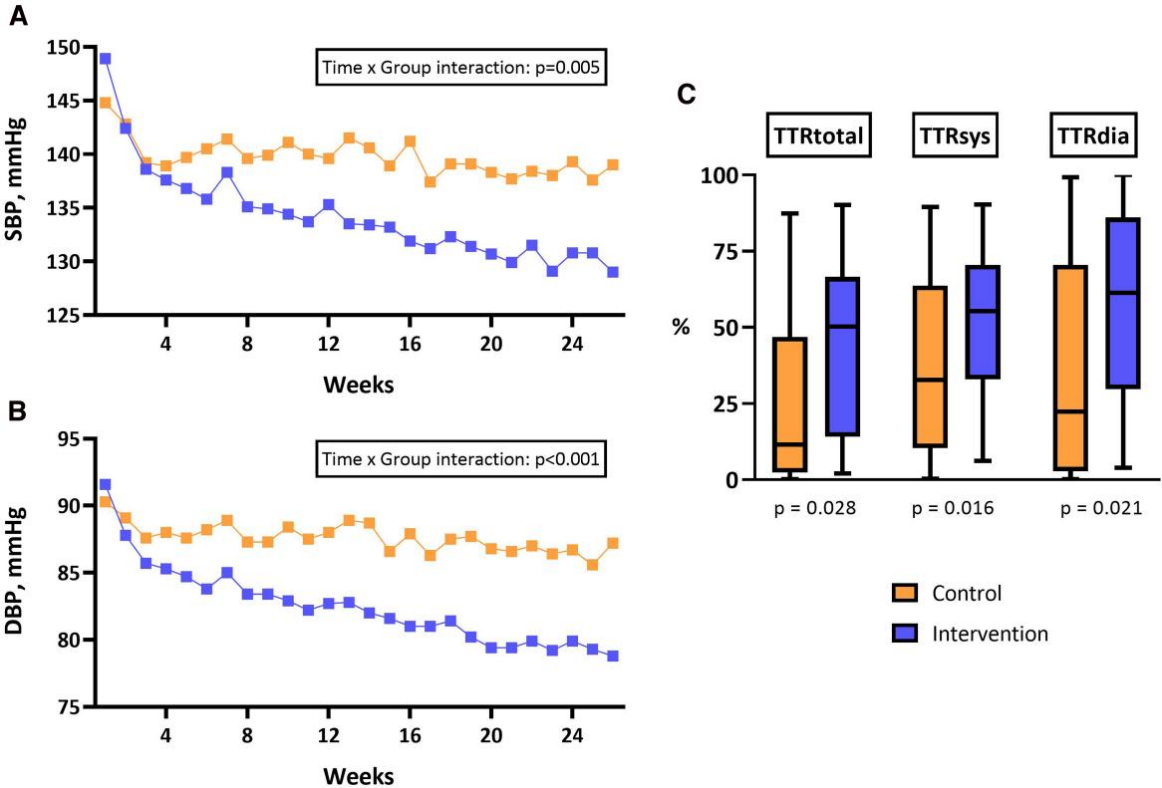
**Blood pressure trajectories and time in target range over 6 months.** This figure displays (*A*) mean weekly systolic blood pressure (SBP) and (*B*) mean weekly diastolic blood pressure (DBP) over the first 26 weeks in the intervention and control groups. Values represent the average of all valid home BP measurements per patient and week. *P*-values for time × group interaction were derived using repeated-measures ANOVA. (*C*) Box plots depict the distribution of total, systolic, and diastolic time in target range (TTR) over 6 months in the intervention and control groups. TTR was calculated as the percentage of home BP measurements within guideline-recommended targets. *P*-values for between-group comparisons were derived using unpaired *t*-tests. Statistical significance was defined as *P* < 0.05.

At 6 months, patients in the telemedical care group demonstrated significantly greater improvements in BP control compared to those receiving standard care (*Table 2*, *Figure 2A–C*). Mean home systolic BP was significantly lower in the intervention than in the control group (134 ± 8 mmHg vs. 140 ± 12 mmHg, *P* = 0.033), with a similar difference in diastolic BP (82 ± 7 mmHg vs. 89 ± 10 mmHg, *P* = 0.006). Heart rate remained comparable between groups (72 ± 9 bpm for both, *P* = 0.924).

**Table 2:** Blood pressure outcomes, medication use, and BP measurement adherence at 6 months

	All patients *n* = 56	Control *n* = 27	Intervention *n* = 29	*P*-value^a^
**Systolic BP, mmHg**	137 ± 10	140 ± 12	134 ± 8	0.033
**Diastolic BP, mmHg**	85 ± 9	89 ± 10	82 ± 7	0.006
**Heart rate, bpm**	72 ± 9	72 ± 8	72 ± 9	0.924
**Total TTR month 0–6, %**	34.2 ± 29.7	24.8 ± 29.7	42.9 ± 27.5	0.021
**Systolic TTR month 0–6, %**	44.4 ± 27.7	36.0 ± 29.2	52.2 ± 24.2	0.028
**Diastolic TTR month 0–6, %**	48.4 ± 34.4	37.1 ± 34.4	59.0 ± 31.0	0.016
**Total TTR at month 6, %**	39.1 ± 32.0	23.5 ± 31.2	53.2 ± 26.0	<0.001
**Systolic TTR at month 6, %**	50.8 ± 29.4	36.6 ± 30.9	63.5 ± 21.4	0.001
**Diastolic TTR at month 6, %**	55.0 ± 36.5	37.5 ± 37.0	70.8 ± 28.2	<0.001
**Mean systolic BP (weeks 1–2), mmHg**	144 ± 11	143 ± 12	144 ± 11	0.725
**Mean systolic BP (weeks 25–26), mmHg**	134 ± 11	139 ± 12	129 ± 7	0.001
**Change in systolic BP (weeks 1–2 to 25–26), mmHg**	−9.8 ± 10.7	−4.0 ± 8.7	−15.0 ± 9.8	<0.001
**Mean diastolic BP (weeks 1–2), mmHg**	89 ± 8	89 ± 8	89 ± 9	0.863
**Mean diastolic BP (weeks 25–26), mmHg**	83 ± 9	87 ± 9	79 ± 7	<0.001
**Change in diastolic BP (weeks 1–2 to 25–26), mmHg**	−6.0 ± 7.3	−1.8 ± 5.6	−9.8 ± 6.7	<0.001
**BP measurement adherence** ^b^ **, %**	77.1 ± 21.9	70.5 ± 25.9	83.2 ± 15.4	0.033
**Number of antihypertensive medication at BL**	2.3 ± 1.0	2.4 ± 0.8	2.3 ± 1.1	0.819
**Number of antihypertensive medication at 6 months**	3.4 ± 1.2	3.2 ± 1.2	3.6 ± 1.2	0.221
**Change in antihypertensive medication from BL to month 6**	1.1 ± 1.2	0.8 ± 1.0	1.3 ± 1.3	0.152

^a^Between-group comparisons were performed using independent-samples *t*-tests. Statistical significance was defined as *P* < 0.05.

^b^Measurement compliance was defined as the proportion of completed BP measurements over the total expected (four measurements per day * study days). Abbreviations: BL, baseline; BP, blood pressure; TTR, time in target range.

BP control over time was assessed using the metric TTR. Over the first 6 months, total TTR was significantly higher in the telemedical care group (42.9 ± 27.5%) compared to standard care (24.8 ± 29.7%, *P* = 0.021; *Figure 2C*). This effect was also evident when analysing systolic (52.2 ± 24.2% vs. 36.0 ± 29.2%, *P* = 0.028) and diastolic TTR separately (59.0 ± 31.0% vs. 37.1 ± 34.4%, *P* = 0.016).

As an exploratory analysis, TTR was assessed for month 6 alone, where the between-group differences were even more pronounced: systolic TTR: 63.5 ± 21.4% vs. 36.6 ± 30.9%, *P* = 0.001; diastolic TTR: 70.8 ± 28.2% vs. 37.5 ± 37.0%, *P* < 0.001; and total TTR: 53.2 ± 26.0% vs. 23.5 ± 31.2%, *P* < 0.001.

Longitudinal analysis of home BP values over 26 weeks revealed three distinct phases of BP change (*Figure 2A + B*). During the initial 4 weeks (Phase 1), BP declined in both groups. This was followed by a diverging trajectory between groups: from week 4 to approximately week 16 (Phase 2), BP in the intervention group continued to decline, whereas BP in the control group remained largely unchanged. In the final phase (weeks 16–26), BP values in the intervention group displayed a slower but continued decline with final stabilization at lower levels, while those in the control group remained at a higher plateau. These differences were confirmed by repeated-measures ANOVA, showing a significant time × group interaction for both systolic (*F* = 3.193, *P* = 0.005) and diastolic BP (*F* = 4.298, *P* < 0.001). Between Weeks 1–2 and Weeks 25–26, mean systolic BP decreased by −15.0 ± 9.8 mmHg in the telemedical group, compared to −4.0 ± 8.7 mmHg in the control group (*P* < 0.001). A similar pattern was observed for diastolic BP (−9.8 ± 6.7 mmHg vs. −1.8 ± 5.6 mmHg, *P* < 0.001), (*Table 2*).

The number of antihypertensive drug classes was comparable between groups at baseline (2.3 ± 1.0 vs. 2.4 ± 0.8, *P* = 0.723) and remained statistically similar at 6 months (3.5 ± 1.2 vs. 3.1 ± 1.2, *P* = 0.169).

Overall adherence to home BP monitoring was also higher in the intervention group (83.2 ± 15.4% vs. 70.5 ± 25.9%, *P* = 0.033).

### Subgroup analysis of time in target range

**Figure 3:**
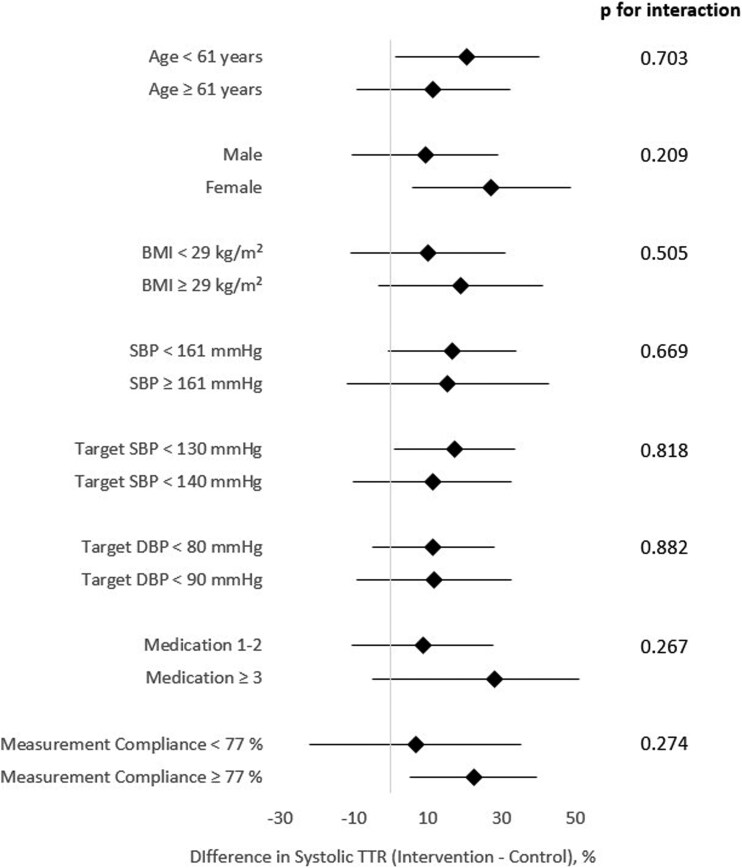
**Subgroup analysis of systolic time in target range.** This forest plot shows the difference in systolic time in target range between intervention and control groups across predefined clinical subgroups. Subgroup cut-offs were based on the respective mean values (see *Table 1*, baseline characteristics). Diamonds represent the mean difference (intervention minus control) and horizontal lines indicate 95% confidence intervals. Positive values indicate a benefit of the intervention. Interaction *P*-values test whether the effect of the intervention differs significantly between subgroups. Abbreviations: BMI, body mass index; DBP, diastolic blood pressure; SBP, systolic blood pressure; TTR, time in target range.

To further characterize the effect of telemedical care on BP control, a subgroup analysis was performed assessing differences in systolic TTR between intervention and control groups across relevant clinical and demographic subgroups (*Figure 3*).

While systolic TTR was consistently higher in the intervention group across all subgroups, the magnitude of difference varied. Notably, the largest absolute improvements were observed in patients <61 years (+20.6%), females (+27.2%), those with baseline systolic BP <161 mmHg (+16.6%), and patients on ≥3 antihypertensive drugs at baseline (+28.1%). However, none of the *P*-values for interaction were statistically significant (all *P* > 0.05), indicating no robust evidence for differential treatment effect across subgroups.

A similar pattern was observed for total and diastolic TTR (see Supplementary material online, *Table S1*). Patients with higher adherence to BP measurements, female sex, and lower baseline BP values again showed numerically larger between-group differences, though interaction testing remained non-significant throughout.

### Safety/adverse events

Overall, at least one side effect was reported in 31 participants (51.7%)—44.8% in the control group vs. 58.1% in the intervention group (*P* = 0.305)—with a total of 49 adverse reactions documented (*Table 3*). Symptomatic hypotension, which was defined as patient-reported symptoms compatible with hypotension (e.g. dizziness, presyncope, or weakness) that prompted clinical review and/or medication adjustment, was numerically more frequent in the intervention group (22.6% vs. 6.9%, *P* = 0.089). Severely elevated home BP readings, predefined as a home BP reading ≥200/120 mmHg, were more common in the control group (12.9% vs. 34.5%, *P* = 0.048). These readings were not adjudicated as hypertensive emergencies and did not require symptoms. Other reported adverse events, such as electrolyte imbalances (5.0% overall), circulatory complaints (6.7% overall), and deterioration in kidney function (3.3% overall), were rare in both groups and with no statistically significant between-group differences. Other, non-specific side effects, not conclusively linked to the medication used, were significantly more frequent in the intervention group (38.7% vs. 6.9%, *P* = 0.030). This included but was not limited to unspecific fatigue, headache, sleep disturbance, or musculoskeletal complaints.

**Table 3:** Adverse events

	All patients *n* = 60	Control *n* = 29	Intervention *n* = 31	*P*-value^a^
**Patients with ≥1 side effect, *n* (%)**	31 (51.7)	13 (44.8)	18 (58.1)	0.305
**Total number of reported side effects**	49	22	27	0.516
**Symptomatic hypotension, *n* (%)**	9 (15.0)	2 (6.9)	7 (22.6)	0.089
**Medical visit due to hypertension, *n* (%)**	4 (6.7)	2 (6.9)	2 (6.5)	>0.999
**Severely elevated home BP readings (≥200/120 mmHg), *n* (%)**	14 (23.3)	10 (34.5)	4 (12.9)	0.048
**Electrolyte imbalance, *n* (%)**	3 (5.0)	1 (3.4)	2 (6.5)	>0.999
**Circulatory complaints, *n* (%)**	4 (6.7)	3 (10.3)	1 (3.2)	0.346
**Deterioration of kidney function, *n* (%)**	2 (3.3)	2 (6.9)	0 (0)	0.221
**Gastrointestinal side effects, *n* (%)**	0 (0)	0 (0)	0 (0)	
**Other side effects, *n* (%)**	14 (23.3)	2 (6.9)	12 (38.7)	0.030

^a^Between-group comparisons were performed by Pearson’s χ^2^ test. For small case numbers (*n* ≤ 5) Fisher’s exact test was used. Statistical significance was defined as *P* < 0.05

## Discussion

In this prospective, randomized pilot trial, structured telemedical care with biweekly physician-led phone consultations significantly improved BP control compared to standard care in patients with uncontrolled arterial hypertension over a 6-month period. Patients receiving telemedical intervention showed significantly higher TTR, accompanied by larger reductions in systolic and diastolic BP, and better adherence to home BP measurements compared to standard care. These findings support the use of telemedical strategies to enhance hypertension management in routine outpatient settings.

We acknowledge that TTR is not yet standardized and that its value can be influenced by the frequency, method of BP measurement, and target range definitions, which may limit comparability with other studies. However, TTR is an emerging metric for assessing BP control that incorporates both BP variability and overall target achievement and has therefore been increasingly recognized as a meaningful metric for cardiovascular risk stratification.^15,16^ In the current study, TTR was significantly higher in the intervention group across all domains. While the overall TTR improvement was substantial, the intervention group showed particularly large benefits in systolic TTR in several clinically relevant subgroups, including younger patients, females, patients with stricter BP targets <130/80 mmHg, and those on ≥3 antihypertensive drug classes at point of inclusion. However, due to the small patient cohort, the subgroup analysis must be regarded carefully.

Some patients in the intervention group required several weeks to achieve normotensive values despite early medication adjustments, some not reaching a normotensive range until month 6. This delay led to a relatively low overall 6-month TTR for these individuals despite successful BP lowering over time. In order to understand this delayed treatment response, we conducted an exploratory, *post hoc* analysis of TTR limited to month 6. This revealed even greater differences between groups, underscoring the progressive benefit of the intervention and highlighting the importance of time-sensitive interpretations of TTR.

In addition, overall adherence to home BP monitoring was significantly higher in the intervention group, which likely facilitated more frequent and tailored medication adjustments. While BP control improved, the total number of antihypertensive drug classes at 6 months did not significantly differ between groups. This may in part be explained by the inclusion of several patients who became normotensive shortly after baseline due to medication optimization at enrollment. These patients remained normotensive throughout the study and therefore did not require further adjustments, contributing to an underestimation of the treatment intensity in the intervention group. Moreover, dose up-titration of pre-existing antihypertensive medication, an important component of treatment intensification, was not captured by the number of drug classes and therefore not reflected in the medication count analysis. Nevertheless, the numerically greater increase in medication count in the intervention group suggests more active management overall, which likely contributed to the superior BP outcomes.

The trajectory of BP improvement over the 26-week period highlights the dynamic nature of hypertension management. Three distinct phases were observed: an initial decline in both groups during the first 4 weeks, a continued decrease in the intervention group from Weeks 4 to 16, and a slower but steady improvement in the final phase (Weeks 16–26), whereas the control group plateaued early and remained largely unchanged. This pattern underscores the sustained benefit of regular physician contact and reinforces the value of long-term engagement for optimal BP control.

Although side effects occurred more frequently in the intervention group, this likely reflects the more intensive treatment strategy and close monitoring rather than an inherent risk of telemedicine.^11,17^ Importantly, the structured follow-up enabled proactive adjustments—typically switching agents within the same class or to a different class—thus avoiding therapy discontinuation without alternatives. Symptoms of intolerance (e.g. dizziness, fatigue) were numerically more common under telemedical care but did not show statistical significance. These often coincided with still-elevated or near-target BP values and were not associated with hypotension *per se*. Temporary adjustments allowed most patients to continue therapy and eventually reach target BP levels. In contrast, significantly more severely elevated home BP readings (≥200/120 mmHg) occurred in the control group, underscoring the potential risk of insufficient follow-up in routine care. While patients performed duplicate measurements and values ≥200 mmHg were typically confirmed by the second reading within the same sitting, technical outliers cannot be fully excluded. While study team alerts led to appropriate follow-up recommendations, such events may have clinical consequences if left unaddressed in real-world settings.^18^

The current findings align with previous studies suggesting that telemedical care can effectively lower BP in patients with hypertension. In TASMINH2, self-monitoring with patient self-titration plus telemonitoring reduced systolic BP vs. usual care at 6 and 12 months.^17^ In TASMINH4, general practitioner-led titration using self-monitored BP lowered BP vs. usual care, but the incremental effect of adding telemonitoring over self-monitoring alone at 12 months was small and not statistically significant.^19^ Smaller nurse-led telenursing trials in prehypertension point in the same direction but have limited generalizability to hypertensive populations.^10^ Our randomized pilot study adds novel granularity by combining biweekly physician-led consultations with protocolized medication adaption, quantifying TTR across domains, demonstrating higher adherence and a three-phase trajectory of BP improvement, and offer comparable BP measurements for the control group. Collectively, these data align with the broader literature that telemedical care can improve BP control when remote data are paired with structured, clinician-driven action. However, while telemedical care demonstrated a significant reduction in cardiovascular mortality and hospitalizations in patients with chronic heart failure,^9^ leading to its reimbursement in several countries, large-scale outcome trials in hypertension remain absent.

Several studies have demonstrated that lowering BP reduces the risk of cardiovascular mortality, and major cardiovascular events.^20–24^ While our pilot trial was not designed to assess hard clinical outcomes, the significant improvement in BP control observed through telemedical intervention suggests a potential for long-term cardiovascular benefit. In a real-world setting, implementations could be facilitated through integration with specialized hypertension clinics, use of telemedicine platforms, and targeted inclusion of patients with difficult-to-control hypertension. Larger, clinical trials are warranted to evaluated the impact of telemedical care on major cardiovascular outcomes, potentially leading to a reimbursement for telemedical care in the field of hypertension and training non-physician healthcare providers.

## Limitations

This single-centre trial enrolled a relatively small sample size (*n* = 60) and was conducted at a specialized tertiary care centre with intervention being delivered by study physicians with high expertise in hypertension management, potentially limiting generalizability. While only four patients formally withdrew consent, a small number of participants could not be contacted regularly despite repeated attempts, which may have reduced intervention intensity. The inclusion criterion of office-BP >140/90 mmHg, based on a single measurement, led to the enrollment of some patients who became normotensive shortly after inclusion—often following minimal medication adjustments—possibly diluting the observed treatment effect. Mandatory 24-h BP monitoring prior to inclusion may have better identified individuals with sustained hypertension. BP targets were individualized based on ESC guideline thresholds at the time of trial design. However, in a real-world application—even with gradual BP reduction through close telemonitoring—target values had to be adjusted in four patients due to intolerance of lower BP values. Finally, the TTR metric may underestimate treatment effects in patients with initially severe hypertension who achieved control only late in the study. When analysing TTR during the final month a delayed but substantial treatment effect in these patients can be observed.

## Conclusion

In this randomized controlled trial, telemedical care with regular physician-led consultations significantly improved BP control and adherence to home BP monitoring in patients with uncontrolled arterial hypertension compared to standard care. These findings support the role of telemonitoring as a tool to enhance outpatient hypertension management. Larger trials are needed to confirm long-term clinical benefits.

## Supplementary Material

oeag061_Supplementary_Data

## Data Availability

Additional data underlying this article cannot be shared publicly due to the privacy of individuals that participated in the study. De-identified data will be shared on reasonable request to the corresponding author.

## References

[1] Zhou B, Carrillo-Larco RM, Danaei G, Riley LM, Paciorek CJ, Stevens GA, Gregg EW, Bennett JE, Solomon B, Singleton RK, Sophiea MK, Iurilli ML, Lhoste VP, Cowan MJ, Savin S, Woodward M, Balanova Y, Cifkova R, Damasceno A, Elliott P, Farzadfar F, He J, Ikeda N, Kengne AP, Khang Y-H, Kim HC, Laxmaiah A, Lin H-H, Margozzini Maira P, Miranda JJ, Neuhauser H, Sundström J, Varghese C, Widyahening IS, Zdrojewski T, Abarca-Gómez L, Abdeen ZA, Abdul Rahim HF, Abu-Rmeileh NM, Acosta-Cazares B, Adams RJ, Aekplakorn W, Afsana K, Afzal S, Agdeppa IA, Aghazadeh-Attari J, Aguilar-Salinas CA, Agyemang C, Ahmad NA, Ahmadi A, Ahmadi N, Ahmadi N, Ahmadizar F, Ahmed SH, Ahrens W, Ajlouni K, Al-Raddadi R, Alarouj M, AlBuhairan F, AlDhukair S, Ali MM, Alkandari A, Alkerwi A, Allin K, Aly E, Amarapurkar DN, Amougou N, Amouyel P, Andersen LB, Anderssen SA, Anjana RM, Ansari-Moghaddam A, Ansong D, Aounallah-Skhiri H, Araújo J, Ariansen I, Aris T, Arku RE, Arlappa N, Aryal KK, Aspelund T, Assah FK, Assunção MCF, Auvinen J, Avdićová M, Azevedo A, Azimi-Nezhad M, Azizi F, Azmin M, Babu BV, Bahijri S, Balakrishna N, Bamoshmoosh M, Banach M, Banadinović M, Bandosz P, Banegas JR, Baran J, Barbagallo CM, Barceló A, Barkat A, Barreto M, Barros AJ, Barros MVG, Bartosiewicz A, Basit A, Bastos JLD, Bata I, Batieha AM, Batyrbek A, Baur LA, Beaglehole R, Belavendra A, Ben Romdhane H, Benet M, Benson LS, Berkinbayev S, Bernabe-Ortiz A, Bernotiene G, Bettiol H, Bezerra J, Bhagyalaxmi A, Bhargava SK, Bia D, Biasch K, Bika Lele EC, Bikbov MM, Bista B, Bjerregaard P, Bjertness E, Bjertness MB, Björkelund C, Bloch KV, Blokstra A, Bo S, Bobak M, Boeing H, Boggia JG, Boissonnet CP, Bojesen SE, Bongard V, Bonilla-Vargas A, Bopp M, Borghs H, Bovet P, Boyer CB, Braeckman L, Brajkovich I, Branca F, Breckenkamp J, Brenner H, Brewster LM, Briceño Y, Brito M, Bruno G, Bueno-de-Mesquita HB, Bueno G, Bugge A, Burns C, Bursztyn M, Cabrera de León A, Cacciottolo J, Cameron C, Can G, Cândido APC, Capanzana MV, Čapková N, Capuano E, Capuano V, Cardoso VC, Carlsson AC, Carvalho J, Casanueva FF, Censi L, Cervantes-Loaiza M, Chadjigeorgiou CA, Chamukuttan S, Chan AW, Chan Q, Chaturvedi HK, Chaturvedi N, Chee ML, Chen C-J, Chen F, Chen H, Chen S, Chen Z, Cheng C-Y, Cheraghian B, Cherkaoui Dekkaki I, Chetrit A, Chien K-L, Chiolero A, Chiou S-T, Chirita-Emandi A, Chirlaque M-D, Cho B, Christensen K, Christofaro DG, Chudek J, Cinteza E, Claessens F, Clarke J, Clays E, Cohen E, Concin H, Cooper C, Coppinger TC, Costanzo S, Cottel D, Cowell C, Craig CL, Crampin AC, Crujeiras AB, Cruz JJ, Csilla S, Cui L, Cureau FV, Cuschieri S, D'Arrigo G, d'Orsi E, Dallongeville J, Dankner R, Dantoft TM, Dauchet L, Davletov K, De Backer G, De Bacquer D, De Curtis A, de Gaetano G, De Henauw S, de Oliveira PD, De Ridder D, De Smedt D, Deepa M, Deev AD, DeGennaro VJ, Delisle H, Demarest S, Dennison E, Deschamps V, Dhimal M, Di Castelnuovo AF, Dias-da-Costa JS, Diaz A, Dickerson TT, Dika Z, Djalalinia S, Do HT, Dobson AJ, Donfrancesco C, Donoso SP, Döring A, Dorobantu M, Dörr M, Doua K, Dragano N, Drygas W, Duante CA, Duboz P, Duda RB, Dulskiene V, Dushpanova A, Džakula A, Dzerve V, Dziankowska-Zaborszczyk E, Eddie R, Eftekhar E, Eggertsen R, Eghtesad S, Eiben G, Ekelund U, El-Khateeb M, El Ati J, Eldemire-Shearer D, Eliasen M, Elosua R, Erasmus RT, Erbel R, Erem C, Eriksen L, Eriksson JG, Escobedo-de la Peña J, Eslami S, Esmaeili A, Evans A, Faeh D, Fakhretdinova AA, Fall CH, Faramarzi E, Farjam M, Fattahi MR, Fawwad A, Felix-Redondo FJ, Felix SB, Ferguson TS, Fernandes RA, Fernández-Bergés D, Ferrante D, Ferrao T, Ferrari M, Ferrario MM, Ferreccio C, Ferreira HS, Ferrer E, Ferrieres J, Figueiró TH, Fink G, Fischer K, Foo LH, Forsner M, Fouad HM, Francis DK, Franco MdC, Frikke-Schmidt R, Frontera G, Fuchs FD, Fuchs SC, Fujita Y, Fumihiko M, Furdela V, Furer A, Furusawa T, Gaciong Z, Galbarczyk A, Galenkamp H, Galvano F, Gao J, Gao P, Garcia-de-la-Hera M, Garcia P, Gareta D, Garnett SP, Gaspoz J-M, Gasull M, Gazzinelli A, Gehring U, Geleijnse JM, George R, Ghanbari A, Ghasemi E, Gheorghe-Fronea O-F, Ghimire A, Gialluisi A, Giampaoli S, Gieger C, Gill TK, Giovannelli J, Gironella G, Giwercman A, Gkiouras K, Goldberg M, Goldsmith RA, Gomez LF, Gomula A, Gonçalves H, Gonçalves M, Gonçalves Cordeiro da Silva B, Gonzalez-Chica DA, Gonzalez-Gross M, González-Rivas JP, González-Villalpando C, González-Villalpando M-E, Gonzalez AR, Gorbea MB, Gottrand F, Graff-Iversen S, Grafnetter D, Grajda A, Grammatikopoulou MG, Gregor RD, Grodzicki T, Grosso G, Gruden G, Gu D, Guan OP, Gudmundsson EF, Gudnason V, Guerrero R, Guessous I, Guimaraes AL, Gulliford MC, Gunnlaugsdottir J, Gunter MJ, Gupta PC, Gupta R, Gureje O, Gurzkowska B, Gutierrez L, Gutzwiller F, Ha S, Hadaegh F, Haghshenas R, Hakimi H, Halkjær J, Hambleton IR, Hamzeh B, Hange D, Hanif AA, Hantunen S, Hao J, Hardman CM, Hari Kumar R, Hashemi-Shahri SM, Hata J, Haugsgjerd T, Hayes AJ, He Y, Heier M, Hendriks ME, Henrique RdS, Henriques A, Hernandez Cadena L, Herrala S, Heshmat R, Hill AG, Ho SY, Ho SC, Hobbs M, Holdsworth M, Homayounfar R, Horasan Dinc G, Horimoto AR, Hormiga CM, Horta BL, Houti L, Howitt C, Htay TT, Htet AS, Htike MMT, Hu Y, Huerta JM, Huhtaniemi IT, Huiart L, Huisman M, Husseini AS, Huybrechts I, Hwalla N, Iacoviello L, Iannone AG, Ibrahim MM, Ibrahim Wong N, Ikram MA, Iotova V, Irazola VE, Ishida T, Isiguzo GC, Islam M, Islam SMS, Iwasaki M, Jackson RT, Jacobs JM, Jaddou HY, Jafar T, James K, Jamrozik K, Janszky I, Janus E, Jarvelin M-R, Jasienska G, Jelaković A, Jelaković B, Jennings G, Jha AK, Jiang CQ, Jimenez RO, Jöckel K-H, Joffres M, Johansson M, Jokelainen JJ, Jonas JB, Jørgensen T, Joshi P, Joukar F, Jóżwiak J, Juolevi A, Jurak G, Jureša V, Kaaks R, Kafatos A, Kajantie EO, Kalmatayeva Z, Kalpourtzi N, Kalter-Leibovici O, Kampmann FB, Kannan S, Karaglani E, Kårhus LL, Karki KB, Katibeh M, Katz J, Kauhanen J, Kaur P, Kavousi M, Kazakbaeva GM, Keil U, Keinan Boker L, Keinänen-Kiukaanniemi S, Kelishadi R, Kemper HC, Keramati M, Kerimkulova A, Kersting M, Key T, Khader YS, Khalili D, Khaw K-T, Kheiri B, Kheradmand M, Khosravi A, Kiechl-Kohlendorfer U, Kiechl S, Killewo J, Kim DW, Kim J, Klakk H, Klimek M, Klumbiene J, Knoflach M, Kolle E, Kolsteren P, Kontto JP, Korpelainen R, Korrovits P, Kos J, Koskinen S, Kouda K, Kowlessur S, Koziel S, Kratenova J, Kriaucioniene V, Kristensen PL, Krokstad S, Kromhout D, Kruger HS, Kubinova R, Kuciene R, Kujala UM, Kulaga Z, Kumar RK, Kurjata P, Kusuma YS, Kutsenko V, Kuulasmaa K, Kyobutungi C, Laatikainen T, Lachat C, Laid Y, Lam TH, Landrove O, Lanska V, Lappas G, Larijani B, Latt TS, Le Coroller G, Le Nguyen Bao K, Le TD, Lee J, Lee J, Lehmann N, Lehtimäki T, Lemogoum D, Levitt NS, Li Y, Lilly CL, Lim W-Y, Lima-Costa MF, Lin X, Lin Y-T, Lind L, Lingam V, Linneberg A, Lissner L, Litwin M, Lo W-C, Loit H-M, Lopez-Garcia E, Lopez T, Lotufo PA, Lozano JE, Lukačević Lovrenčić I, Lukrafka JL, Luksiene D, Lundqvist A, Lundqvist R, Lunet N, Lustigová M, Luszczki E, Ma G, Ma J, Machado-Coelho GL, Machado-Rodrigues AM, Macia E, Macieira LM, Madar AA, Maggi S, Magliano DJ, Magriplis E, Mahasampath G, Maire B, Majer M, Makdisse M, Malekzadeh F, Malekzadeh R, Malhotra R, Mallikharjuna Rao K, Malyutina SK, Maniego LV, Manios Y, Mann JI, Mansour-Ghanaei F, Manzato E, Marcil A, Mårild SB, Marinović Glavić M, Marques-Vidal P, Marques LP, Marrugat J, Martorell R, Mascarenhas LP, Matasin M, Mathiesen EB, Mathur P, Matijasevich A, Matlosz P, Matsha TE, Mavrogianni C, Mbanya JCN, Mc Donald Posso AJ, McFarlane SR, McGarvey ST, McLachlan S, McLean RM, McLean SB, McNulty BA, Mediene Benchekor S, Medzioniene J, Mehdipour P, Mehlig K, Mehrparvar AH, Meirhaeghe A, Meisinger C, Mendoza Montano C, Menezes AMB, Menon GR, Mereke A, Meshram II, Metspalu A, Meyer HE, Mi J, Michels N, Mikkel K, Milkowska K, Miller JC, Minderico CS, Mini GK, Mirjalili MR, Mirrakhimov E, Mišigoj-Duraković M, Modesti PA, Moghaddam SS, Mohajer B, Mohamed MK, Mohamed SF, Mohammad K, Mohammadi MR, Mohammadi Z, Mohammadifard N, Mohammadpourhodki R, Mohan V, Mohanna S, Mohd Yusoff MF, Mohebbi I, Mohebi F, Moitry M, Møllehave LT, Molnár D, Momenan A, Mondo CK, Monterrubio-Flores E, Monyeki KDK, Moon JS, Moosazadeh M, Moreira LB, Morejon A, Moreno LA, Morgan K, Moschonis G, Mossakowska M, Mostafa A, Mostafavi S-A, Mota J, Motlagh ME, Motta J, Moura-dos-Santos MA, Mridha MK, Msyamboza KP, Mu TT, Muhihi AJ, Muiesan ML, Müller-Nurasyid M, Murphy N, Mursu J, Musa KI, Musić Milanović S, Musil V, Mustafa N, Nabipour I, Naderimagham S, Nagel G, Naidu BM, Najafi F, Nakamura H, Námešná J, Nang EEK, Nangia VB, Narake S, Ndiaye NC, Neal WA, Nejatizadeh A, Nenko I, Neovius M, Nguyen CT, Nguyen ND, Nguyen QV, Nguyen QN, Nieto-Martínez RE, Niiranen TJ, Nikitin YP, Ninomiya T, Nishtar S, Njelekela MA, Noale M, Noboa OA, Noorbala AA, Norat T, Nordendahl M, Nordestgaard BG, Noto D, Nowak-Szczepanska N, Nsour MA, Nunes B, O'Neill TW, O'Reilly D, Ochimana C, Oda E, Odili AN, Oh K, Ohara K, Ohtsuka R, Olié V, Olinto MTA, Oliveira IO, Omar MA, Onat A, Ong SK, Ono LM, Ordunez P, Ornelas R, Ortiz PJ, Osmond C, Ostojic SM, Ostovar A, Otero JA, Overvad K, Owusu-Dabo E, Paccaud FM, Padez C, Pahomova E, Paiva KMd, Pająk A, Palli D, Palmieri L, Pan W-H, Panda-Jonas S, Panza F, Paoli M, Papandreou D, Park S-W, Park S, Parnell WR, Parsaeian M, Pasquet P, Patel ND, Pavlyshyn H, Pećin I, Pednekar MS, Pedro JM, Peer N, Peixoto SV, Peltonen M, Pereira AC, Peres KG, Peres MA, Peters A, Petkeviciene J, Peykari N, Pham ST, Pichardo RN, Pigeot I, Pikhart H, Pilav A, Pilotto L, Pitakaka F, Piwonska A, Pizarro An, Plans-Rubió P, Polašek O, Porta M, Poudyal A, Pourfarzi F, Pourshams A, Poustchi H, Pradeepa R, Price AJ, Price JF, Providencia R, Puhakka SE, Puiu M, Punab M, Qasrawi RF, Qorbani M, Queiroz D, Quoc Bao T, Radić I, Radisauskas R, Rahimikazerooni S, Rahman M, Raitakari O, Raj M, Rakhimova EM, Ramachandra Rao S, Ramachandran A, Ramos E, Rampal L, Rampal S, Rangel Reina DA, Rarra V, Rech CR, Redon J, Reganit PFM, Regecová V, Revilla L, Rezaianzadeh A, Ribeiro R, Riboli E, Richter A, Rigo F, Rinke de Wit TF, Ritti-Dias RM, Robitaille C, Rodríguez-Artalejo F, Rodriguez-Perez MdC, Rodríguez-Villamizar LA, Roggenbuck U, Rojas-Martinez R, Romaguera D, Romeo EL, Rosengren A, Roy JG, Rubinstein A, Ruidavets J-B, Ruiz-Betancourt BS, Ruiz-Castell M, Rusakova IA, Russo P, Rutkowski M, Sabanayagam C, Sabbaghi H, Sachdev HS, Sadjadi A, Safarpour AR, Safi S, Safiri S, Saidi O, Sakarya S, Saki N, Salanave B, Salazar Martinez E, Salmerón D, Salomaa V, Salonen JT, Salvetti M, Sánchez-Abanto J, Sans S, Santos DA, Santos IS, Santos LC, Santos MP, Santos R, Saramies JL, Sardinha LB, Sarganas G, Sarrafzadegan N, Sathish T, Saum K-U, Savva S, Sawada N, Sbaraini M, Scazufca M, Schaan BD, Schargrodsky H, Schipf S, Schmidt CO, Schnohr P, Schöttker B, Schramm S, Schultsz C, Schutte AE, Sebert S, Sein AA, Sen A, Senbanjo IO, Sepanlou SG, Servais J, Shalnova SA, Shamah-Levy T, Shamshirgaran M, Shanthirani CS, Sharafkhah M, Sharma SK, Shaw JE, Shayanrad A, Shayesteh AA, Shi Z, Shibuya K, Shimizu-Furusawa H, Shin DW, Shirani M, Shiri R, Shrestha N, Si-Ramlee K, Siani A, Siantar R, Sibai AM, Silva CRdM, Silva DAS, Simon M, Simons J, Simons LA, Sjöström M, Slowikowska-Hilczer J, Slusarczyk P, Smeeth L, So H-K, Soares FC, Sobngwi E, Söderberg S, Soemantri A, Sofat R, Solfrizzi V, Somi MH, Sonestedt E, Song Y, Sørensen TI, Sørgjerd EP, Sorić M, Sossa Jérome C, Soumaré A, Sparboe-Nilsen B, Sparrenberger K, Staessen JA, Starc G, Stavreski B, Steene-Johannessen J, Stehle P, Stein AD, Stergiou GS, Stessman J, Stieber J, Stöckl D, Stocks T, Stokwiszewski J, Stronks K, Strufaldi MW, Suka M, Sun C-A, Sung Y-T, Suriyawongpaisal P, Sy RG, Syddall HE, Sylva RC, Szklo M, Tai ES, Tammesoo M-L, Tamosiunas A, Tan EJ, Tang X, Tanser F, Tao Y, Tarawneh MR, Tarqui-Mamani CB, Taylor A, Taylor J, Tebar WR, Tell GS, Tello T, Tham YC, Thankappan KR, Theobald H, Theodoridis X, Thijs L, Thinggaard M, Thomas N, Thorand B, Thuesen BH, Timmermans EJ, Tjandrarini DH, Tjonneland A, Toft U, Tolonen HK, Tolstrup JS, Topbas M, Topór-Madry R, Tormo MJ, Tornaritis MJ, Torrent M, Torres-Collado L, Touloumi G, Traissac P, Triantafyllou A, Trichopoulos D, Trichopoulou A, Trinh OT, Trivedi A, Tshepo L, Tsugane S, Tuliakova AM, Tulloch-Reid MK, Tullu F, Tuomainen T-P, Tuomilehto J, Turley ML, Twig G, Tynelius P, Tzourio C, Ueda P, Ugel E, Ulmer H, Uusitalo HM, Valdivia G, Valvi D, van Dam RM, van den Born B-J, Van der Heyden J, van der Schouw YT, Van Herck K, Van Minh H, Van Schoor NM, van Valkengoed IG, van Zutphen EM, Vanderschueren D, Vanuzzo D, Varbo A, Vasan SK, Vega T, Veidebaum T, Velasquez-Melendez G, Veronesi G, Verschuren WM, Verstraeten R, Victora CG, Viet L, Villalpando S, Vineis P, Vioque J, Virtanen JK, Visvikis-Siest S, Viswanathan B, Vlasoff T, Vollenweider P, Voutilainen A, Wade AN, Walton J, Wambiya EO, Wan Bebakar WM, Wan Mohamud WN, Wanderley Júnior RdS, Wang M-D, Wang N, Wang Q, Wang X, Wang YX, Wang Y-W, Wannamethee SG, Wareham N, Wei W, Weres A, Werner B, Whincup PH, Widhalm K, Wiecek A, Wilks RJ, Willeit J, Willeit P, Williams EA, Wilsgaard T, Wojtyniak B, Wong-McClure RA, Wong A, Wong TY, Woo J, Wu FC, Wu S, Wyszynska J, Xu H, Xu L, Yaacob NA, Yan W, Yang L, Yang X, Yang Y, Yasuharu T, Ye X, Yiallouros PK, Yoosefi M, Yoshihara A, You S-L, Younger-Coleman NO, Yusoff AF, Zainuddin AA, Zakavi SR, Zamani F, Zambon S, Zampelas A, Zapata ME, Zaw KK, Zejglicova K, Zeljkovic Vrkic T, Zeng Y, Zhang L, Zhang Z-Y, Zhao D, Zhao M-H, Zhen S, Zheng Y, Zholdin B, Zhu D, Zins M, Zitt E, Zocalo Y, Zoghlami N, Zuñiga Cisneros J, Ezzati M. Worldwide trends in hypertension prevalence and progress in treatment and control from 1990 to 2019: a pooled analysis of 1201 population-representative studies with 104 million participants. Lancet 2021;398:957–980. 34450083 10.1016/S0140-6736(21)01330-1 PMC8446938

[2] Zhou B, Danaei G, Stevens GA, Bixby H, Taddei C, Carrillo-Larco RM, Solomon B, Riley LM, Di Cesare M, Iurilli MLC, Rodriguez-Martinez A, Zhu A, Hajifathalian K, Amuzu A, Banegas JR, Bennett JE, Cameron C, Cho Y, Clarke J, Craig CL, Cruz JJ, Gates L, Giampaoli S, Gregg EW, Hardy R, Hayes AJ, Ikeda N, Jackson RT, Jennings G, Joffres M, Khang Y-H, Koskinen S, Kuh D, Kujala UM, Laatikainen T, Lehtimäki T, Lopez-Garcia E, Lundqvist A, Maggi S, Magliano DJ, Mann JI, McLean RM, McLean SB, Miller JC, Morgan K, Neuhauser HK, Niiranen TJ, Noale M, Oh K, Palmieri L, Panza F, Parnell WR, Peltonen M, Raitakari O, Rodríguez-Artalejo F, Roy JG, Salomaa V, Sarganas G, Servais J, Shaw JE, Shibuya K, Solfrizzi V, Stavreski B, Tan EJ, Turley ML, Vanuzzo D, Viikari-Juntura E, Weerasekera D, Ezzati M. Long-term and recent trends in hypertension awareness, treatment, and control in 12 high-income countries: an analysis of 123 nationally representative surveys. Lancet 2019;394:639–651. 31327564 10.1016/S0140-6736(19)31145-6 PMC6717084

[3] Neuhauser H, Thamm M, Ellert U. Blood pressure in Germany 2008–2011: results of the German health interview and examination survey for adults (DEGS1). [blutdruck in deutschland 2008–2011 : ergebnisse der studie zur gesundheit erwachsener in deutschland (DEGS1)]. Bundesgesundheitsblatt Gesundheitsforschung Gesundheitsschutz 2013;56:795–801. 23703500 10.1007/s00103-013-1669-6

[4] Mills KT, Bundy JD, Kelly TN, Reed JE, Kearney PM, Reynolds K, Chen J, He J. Global disparities of hypertension prevalence and control: a systematic analysis of population-based studies from 90 countries. Circulation 2016;134:441–450. 27502908 10.1161/CIRCULATIONAHA.115.018912 PMC4979614

[5] McEvoy JW, McCarthy CP, Bruno RM, Brouwers S, Canavan MD, Ceconi C, Christodorescu RM, Daskalopoulou SS, Ferro CJ, Gerdts E, Hanssen H, Harris J, Lauder L, McManus RJ, Molloy GJ, Rahimi K, Regitz-Zagrosek V, Rossi GP, Sandset EC, Scheenaerts B, Staessen JA, Uchmanowicz I, Volterrani M, Touyz RM, Abreu A, Olsen MH, Ambrosetti M, Androulakis E, Bang LE, Bech JN, Borger MA, Boutouyrie P, Bronze L, Buccheri S, Dalmau R, De Pablo Zarzosa MC, Delles C, Fiuza MM, Gabulova R, Haugen BO, Heiss C, Ibanez B, James S, Kapil V, Kayikçioglu M, Køber L, Koskinas KC, Locati ET, MacDonald S, Mihailidou AS, Mihaylova B, Mindham R, Mortensen MB, Nardai S, Neubeck L, Nielsen JC, Nilsson PM, Pasquet AA, Pedro MM, Prescott E, Rakisheva A, Rietzschel E, Rocca B, Rossello X, Schmid J-P, Shantsila E, Sudano I, Timóteo AT, Tsivgoulis G, Ungar A, Vaartjes I, Visseren F, Voeller H, Vrints C, Witkowski A, Zennaro M-C, Zeppenfeld K, Shuka N, Laredj N, Pavo N, Mirzoyev U, van de Borne P, Sokolović Š, Postadzhiyan A, Samardzic J, Agathangelou P, Widimsky J, Olsen MH, El-Kilany WM, Pauklin P, Laukkanen JA, Boulestreau R, Tsinamdzgvrishvili B, Kintscher U, Marketou M, Páll D, Hrafnkelsdóttir ÞJ, Dolan E, Wolak T, Bilo G, Tundybayeva MK, Mirrakhimov E, Trusinskis K, Kiwan G, Msalem O, Badarienė J, Banu C-A, Balbi MM, Caraus A, Boskovic A, Mouine N, Vromen T, Bosevski M, Midtbø HB, Doroszko A, Dores H, Badila E, Bini R, Simić DV, Fras Z, Mazón P, Spaak J, Burkard T, Barakat E, Abdessalem S, Gunes Y, Sirenko YM, Brady AJB, Khamidullaeva GA. 2024 ESC guidelines for the management of elevated blood pressure and hypertension. Eur Heart J 2024;45:3912–4018. 39210715 10.1093/eurheartj/ehae178

[6] Blumenthal JA, Hinderliter AL, Smith PJ, Mabe S, Watkins LL, Craighead L, Ingle K, Tyson C, Lin P-H, Kraus WE, Liao L, Sherwood A. Effects of lifestyle modification on patients with resistant hypertension: results of the TRIUMPH randomized clinical trial. Circulation 2021;144:1212–1226. 34565172 10.1161/CIRCULATIONAHA.121.055329 PMC8511053

[7] Vrijens B, Vincze G, Kristanto P, Urquhart J, Burnier M. Adherence to prescribed antihypertensive drug treatments: longitudinal study of electronically compiled dosing histories. BMJ 2008;336:1114–1117. 18480115 10.1136/bmj.39553.670231.25 PMC2386633

[8] Omboni S, McManus RJ, Bosworth HB, Chappell LC, Green BB, Kario K, Logan AG, Magid DJ, Mckinstry B, Margolis KL, Parati G, Wakefield BJ. Evidence and recommendations on the use of telemedicine for the management of arterial hypertension: an international expert position paper. Hypertension 2020;76:1368–1383. 32921195 10.1161/HYPERTENSIONAHA.120.15873

[9] Koehler F, Koehler K, Deckwart O, Prescher S, Wegscheider K, Kirwan B-A, Winkler S, Vettorazzi E, Bruch L, Oeff M, Zugck C, Doerr G, Naegele H, Störk S, Butter C, Sechtem U, Angermann C, Gola G, Prondzinsky R, Edelmann F, Spethmann S, Schellong SM, Schulze PC, Bauersachs J, Wellge B, Schoebel C, Tajsic M, Dreger H, Anker SD, Stangl K. Efficacy of telemedical interventional management in patients with heart failure (TIM-HF2): a randomised, controlled, parallel-group, unmasked trial. Lancet 2018;392:1047–1057. 30153985 10.1016/S0140-6736(18)31880-4

[10] Sadeghi-Gandomani H, Habibi Z, Eghbali-Babadi M, Khosravi A. Impact of telenursing on blood pressure and body mass Index of people with prehypertension: a randomized controlled clinical trial. Iran J Nurs Midwifery Res 2021;26:544–549. 34900655 10.4103/ijnmr.IJNMR_113_19 PMC8607889

[11] Margolis KL, Asche SE, Bergdall AR, Dehmer SP, Groen SE, Kadrmas HM, Kerby TJ, Klotzle KJ, Maciosek MV, Michels RD, O’Connor PJ, Pritchard RA, Sekenski JL, Sperl-Hillen JM, Trower NK. Effect of home blood pressure telemonitoring and pharmacist management on blood pressure control: a cluster randomized clinical trial. JAMA 2013;310:46–56. 23821088 10.1001/jama.2013.6549 PMC4311883

[12] Uhlig K, Patel K, Ip S, Kitsios GD, Balk EM. Self-measured blood pressure monitoring in the management of hypertension: a systematic review and meta-analysis. Ann Intern Med 2013;159:185–194. 23922064 10.7326/0003-4819-159-3-201308060-00008

[13] Reshetnik A, Gohlisch C, Zidek W, Tölle M, van der Giet M. Validation of the tel-O-GRAPH, a new oscillometric blood pressure-measuring device, according to the British hypertension society protocol. Blood Press Monit 2016;21:307–309. 27096901 10.1097/MBP.0000000000000195

[14] Williams B, Mancia G, Spiering W, Rosei A, Azizi E, Burnier M, Clement M, Coca DL, Simone G de A, Dominiczak A, Kahan T, Mahfoud F, Redon J, Ruilope L, Zanchetti A, Kerins M, Kjeldsen SE, Kreutz R, Laurent S, Lip GYH, McManus R, Narkiewicz K, Ruschitzka F, Schmieder RE, Shlyakhto E, Tsioufis C, Aboyans V, Desormais I, De Backer G, Heagerty AM, Agewall S, Bochud M, Borghi C, Boutouyrie P, Brguljan J, Bueno H, Caiani EG, Carlberg B, Chapman N, Cífková R, Cleland JGF, Collet J-P, Coman IM, de Leeuw PW, Delgado V, Dendale P, Diener H-C, Dorobantu M, Fagard R, Farsang C, Ferrini M, Graham IM, Grassi G, Haller H, Hobbs FDR, Jelakovic B, Jennings C, Katus HA, Kroon AA, Leclercq C, Lovic D, Lurbe E, Manolis AJ, McDonagh TA, Messerli F, Muiesan ML, Nixdorff U, Olsen MH, Parati G, Perk J, Piepoli MF, Polonia J, Ponikowski P, Richter DJ, Rimoldi SF, Roffi M, Sattar N, Seferovic PM, Simpson IA, Sousa-Uva M, Stanton AV, van de Borne P, Vardas P, Volpe M, Wassmann S, Windecker S, Zamorano JL, Windecker S, Aboyans V, Agewall S, Barbato E, Bueno H, Coca A, Collet J-P, Coman IM, Dean V, Delgado V, Fitzsimons D, Gaemperli O, Hindricks G, Iung B, Jüni P, Katus HA, Knuuti J, Lancellotti P, Leclercq C, McDonagh TA, Piepoli MF, Ponikowski P, Richter DJ, Roffi M, Shlyakhto E, Simpson IA, Sousa-Uva M, Zamorano JL, Tsioufis C, Lurbe E, Kreutz R, Bochud M, Rosei EA, Jelakovic B, Azizi M, Januszewics A, Kahan T, Polonia J, van de Borne P, Williams B, Borghi C, Mancia G, Parati G, Clement DL, Coca A, Manolis A, Lovic D, Benkhedda S, Zelveian P, Siostrzonek P, Najafov R, Pavlova O, De Pauw M, Dizdarevic-Hudic L, Raev D, Karpettas N, Linhart A, Olsen MH, Shaker AF, Viigimaa M, Metsärinne K, Vavlukis M, Halimi J-M, Pagava Z, Schunkert H, Thomopoulos C, Páll D, Andersen K, Shechter M, Mercuro G, Bajraktari G, Romanova T, Trušinskis K, Saade GA, Sakalyte G, Noppe S, DeMarco DC, Caraus A, Wittekoek J, Aksnes TA, Jankowski P, Polonia J, Vinereanu D, Baranova EI, Foscoli M, Dikic AD, Filipova S, Fras Z, Bertomeu-Martínez V, Carlberg B, Burkard T, Sdiri W, Aydogdu S, Sirenko Y, Brady A, Weber T, Lazareva I, Backer TD, Sokolovic S, Jelakovic B, Widimsky J, Viigimaa M, Pörsti I, Denolle T, Krämer BK, Stergiou GS, Parati G, Trušinskis K, Miglinas M, Gerdts E, Tykarski A, de Carvalho Rodrigues M, Dorobantu M, Chazova I, Lovic D, Filipova S, Brguljan J, Segura J, Gottsäter A, Pechère-Bertschi A, Erdine S, Sirenko Y, Brady A. 2018 ESC/ESH guidelines for the management of arterial hypertension. Eur Heart J 2018;39:3021–3104. 30165516 10.1093/eurheartj/ehy339

[15] Li W, Gnanenthiran SR, Schutte AE, Tan I. Blood pressure time at target and its prognostic value for cardiovascular outcomes: a scoping review. Hypertens Res 2024;47:2337–2350. 39014114 10.1038/s41440-024-01798-1 PMC11374670

[16] Fatani N, Dixon DL, van Tassell BW, Fanikos J, Buckley LF. Systolic blood pressure time in target range and cardiovascular outcomes in patients with hypertension. J Am Coll Cardiol 2021;77:1290–1299. 33706870 10.1016/j.jacc.2021.01.014 PMC7959178

[17] McManus RJ, Mant J, Bray EP, Holder R, Jones MI, Greenfield S, Kaambwa B, Banting M, Bryan S, Little P, Williams B, Hobbs FDR. Telemonitoring and self-management in the control of hypertension (TASMINH2): a randomised controlled trial. Lancet 2010;376:163–172. 20619448 10.1016/S0140-6736(10)60964-6

[18] Miller JB, Hrabec D, Krishnamoorthy V, Kinni H, Brook RD. Evaluation and management of hypertensive emergency. BMJ 2024;386:e077205. 39059997 10.1136/bmj-2023-077205

[19] McManus RJ, Mant J, Franssen M, Nickless A, Schwartz C, Hodgkinson J, Bradburn P, Farmer A, Grant S, Greenfield SM, Heneghan C, Jowett S, Martin U, Milner S, Monahan M, Mort S, Ogburn E, Perera-Salazar R, Shah SA, Yu L-M, Tarassenko L, Hobbs FDR, Bradley B, Lovekin C, Judge D, Castello L, Dawson M, Brice R, Dunbabin B, Maslen S, Rutter H, Norris M, French L, Loynd M, Whitbread P, Saldana Ortaga L, Noel I, Madronal K, Timmins J, Bradburn P, Hughes L, Hinks B, Bailey S, Read S, Weston A, Spannuth S, Maiden S, Chermahini M, McDonald A, Rajan S, Allen S, Deboys B, Fell K, Johnson J, Jung H, Lister R, Osborne R, Secker A, Qasim I, William K, Harris A, Zhao S, Butcher E, Darbyshire P, Joshi S, Davies J, Talbot C, Hoverd E, Field L, Adcock T, Rooney J, Cooter N, Butler A, Allen N, Abdul-Wahab M, McNicholas K, Peniket L, Dodd K, Mugurza J, Baskerville R, Syed R, Bailey C, Adams J, Uglow P, Townsend N, Macleod A, Hawkins C, Behura S, Crawshaw J, Fox R, Doski W, Aylward M, A'Court C, Rapley D, Walsh J, Batra P, Seoane A, Mukherjee S, Dixon J, Arthur P, Sutcliffe K, Paschallides C, Woof R, Winfrey P, Clark M, Kamali R, Thomas P, Ebbs D, Mather L, Beattie A, Ladha K, Smondulak L, Jemahl S, Hickson P, Stevens L, Crockett T, Shukla D, Binnian I, Vinson P, DeKare-Silver N, Patel R, Singh I, Lumley L, Williams G, Webb M, Bambrough J, Shah N, Dosanjh H, Spannuth F, Paul C, Ganesegaram J, Pike L, Maheswaran V, Paruk F, Ford S, Verma V, Milne K, Lockhat F, Ferguson J, Quirk A-M, Wilson H, Copping D, Bajallan S, Tanvir S, Khan F, Alderson T, Ali A, Young R, Chauhan U, Crockett L, McGovern L, Cubitt C, Weatherill S, Tabassum A, Saunders P, Chauhan N, Johnson S, Walsh J, Marok I, Sharma R, Lumb W, Tweedale J, Smith I, Miller L, Ahmed T, Sanderson M, Jones C, Stokell P, Edwards MJ, Askey A, Spencer J, Morgan K, Knox K, Baker R, Fisher C, Halstead R, Modha N, Buckley D, Stokell C, McCabe JG, Taylor J, Nutbeam H, Smith R, MacGregor C, Davies S, Lindsey M, Cartwright S, Whittle J, Colclough J, Crumbie A, Thomas N, Premchand V, Hamid R, Ali Z, Ward J, Pinney P, Thurston S, Banerjee T. Efficacy of self-monitored blood pressure, with or without telemonitoring, for titration of antihypertensive medication (TASMINH4): an unmasked randomised controlled trial. Lancet 2018;391:949–959. 29499873 10.1016/S0140-6736(18)30309-X PMC5854463

[20] Wright JT Jr, Williamson JD, Whelton PK, Snyder JK, Sink KM, Rocco MV, Reboussin DM, Rahman M, Oparil S, Lewis CE, Kimmel PL, Johnson KC, Goff DC Jr, Fine LJ, Cutler JA, Cushman WC, Cheung AK, Ambrosius WT. A randomized trial of intensive versus standard blood-pressure control. N Engl J Med 2015;373:2103–2116. 26551272 10.1056/NEJMoa1511939 PMC4689591

[21] Yusuf S, Sleight P, Pogue J, Bosch J, Davies R, Dagenais G. Effects of an angiotensin-converting-enzyme inhibitor, ramipril, on cardiovascular events in high-risk patients. N Engl J Med 2000;342:145–153. 10639539 10.1056/NEJM200001203420301

[22] Patel A, MacMahon S, Chalmers J, Neal B, Woodward M, Billot L, Harrap S, Poulter N, Marre M, Cooper M, Glasziou P, Grobbee DE, Hamet P, Heller S, Liu LS, Mancia G, Mogensen CE, Pan CY, Rodgers A, Williams B. Effects of a fixed combination of perindopril and indapamide on macrovascular and microvascular outcomes in patients with type 2 diabetes mellitus (the ADVANCE trial): a randomised controlled trial. Lancet 2007;370:829–840. 17765963 10.1016/S0140-6736(07)61303-8

[23] PROGRESS Collaborative Group . Randomised trial of a perindopril-based blood-pressure-lowering regimen among 6,105 individuals with previous stroke or transient ischaemic attack. Lancet 2001;358:1033–1041. 11589932 10.1016/S0140-6736(01)06178-5

[24] Doumas M, Tsioufis C, Fletcher R, Amdur R, Faselis C, Papademetriou V. Time in therapeutic range, as a determinant of all-cause mortality in patients with hypertension. J Am Heart Assoc 2017;6:e007131. 29101118 10.1161/JAHA.117.007131 PMC5721788

