# Supplementary material for: Physician-led telemedical care enhances blood pressure control in hypertension: a randomized-controlled pilot study (REMOTE-control-HTN)
Source: Eur Heart J Open. 2026 Apr 21;6(2):oeag061. doi: 10.1093/ehjopen/oeag061 (PMC13126044; doi:10.1093/ehjopen/oeag061)
Supplement: oeag061_Supplementary_Data [file oeag061_supplementary_data.docx]

**Supp. Table 1:** Subgroup analysis of Time in Target Range over 6 months.

|  | **TTR total** | | | | **TTR systolic** | | | | **TTR diastolic** | | | |
| --- | --- | --- | --- | --- | --- | --- | --- | --- | --- | --- | --- | --- |
|  | **TTRdiff, %** | **95%-CI** | **p-value^*^** | **p-for-interaction** | **TTRdiff, %** | **95%-CI** | **p-value^*^** | **p-for-interaction** | **TTRdiff, %** | **95%-CI** | **p-value^*^** | **p-for-interaction** |
| **Age <61 y**^†^  (n=26) | 20.1 | 2.0;38.2 | 0.031 | 0.703 | 20.6 | 1.1;40.0 | 0.039 | 0.520 | 26.6 | 5.7;47.5 | 0.015 | 0.472 |
| **Age ≥61y**^†^  (n=30) | 14.8 | -6.5;36.1 | 0.166 |  | 11.5 | -9.4;32.3 | 0.268 |  | 15.7 | -6.8;38.1 | 0.164 |  |
| **Male**  (n=34) | 10.2 | -10.7;31.1 | 0.327 | 0.209 | 9.3 | -10.6;29.1 | 0.349 | 0.227 | 13.4 | -10.1;36.9 | 0.253 | 0.241 |
| **Female**  (n=22) | 30.2 | 6.5;53.9 | 0.015 |  | 27.2 | 5.9;48.6 | 0.015 |  | 34.8 | 6.1;63.5 | 0.02 |  |
| **BMI <29 kg/m²**^†^  (n=29) | 11.6 | -9.9;33.1 | 0.278 | 0.505 | 9.9 | -11.0;30.8 | 0.338 | 0.551 | 17.8 | -9.5;45.1 | 0.192 | 0.744 |
| **BMI ≥29 kg/m²**^†^  (n=27) | 22.4 | -2.8;47.5 | 0.079 |  | 18.8 | -3.4;41.0 | 0.093 |  | 23.8 | -2.3;50.0 | 0.072 |  |
| **SBP <161 mmHg**^†^  (n=35) | 20.6 | 1.5;39.6 | 0.035 | 0.669 | 16.6 | -0.9;34.0 | 0.062 | 0.937 | 32.0 | 9.8;54.3 | 0.006 | 0.126 |
| **SBP ≥161 mmHg**^†^  (n=21) | 13.8 | -13.8;41.3 | 0.309 |  | 15.4 | -12.1;42.8 | 0.255 |  | 4.7 | -23.7;33.0 | 0.734 |  |
| **Target-SBP <130 mmHg**  (n=33) | 17.4 | 3.2;31.6 | 0.018 | 0.818 | 17.3 | 0.8;33.8 | 0.040 | 0.639 | 23.9 | 7.5;40.3 | 0.006 | 0.396 |
| **Target-SBP <140 mmHg**  (n=23) | 14.8 | 4.8;34.3 | 0.131 |  | 11.3 | -10.2;32.8 | 0.280 |  | 13.4 | -5.0;31.7 | 0.144 |  |
| **Target-DBP <80 mmHg**  (n=30) | 11.2 | -2.4;24.7 | 0.102 | 0.882 | 11.5 | -5.1;28.0 | 0.167 | 0.984 | 19.6 | 2.4;36.9 | 0.027 | 0.367 |
| **Target-DBP <90 mmHg**  (n=26) | 12.8 | -7.3;32.9 | 0.193 |  | 11.7 | -9.2;32.7 | 0.248 |  | 8.6 | -9.2;26.3 | 0.328 |  |
| **Medication 1-2**^†^  (n=33) | 10.9 | -9.4;31.2 | 0.281 | 0.267 | 8.6 | -10.5;27.7 | 0.364 | 0.185 | 14.6 | -9.8;38.9 | 0.231 | 0.331 |
| **Medication ≥3**^†^  (n=23) | 28.5 | 3.1;53.9 | 0.029 |  | 28.1 | -5.2;51.1 | 0.019 |  | 32.3 | 4.8;59.9 | 0.023 |  |
| **Compliance <77 %**^†^  (n=21) | 6.2 | -24.3;36.6 | 0.677 | 0.274 | 6.6 | -22.2;35.4 | 0.638 | 0.302 | 5.7 | -28.3;39.8 | 0.729 | 0.278 |
| **Compliance ≥77%**^†^  (n=35) | 24.1 | 5.6;42.7 | 0.012 |  | 22.5 | 5.3;39.7 | 0.012 |  | 25.6 | 5.1;46.0 | 0.016 |  |

* Between-group comparisons were performed using independent-samples t-tests. Statistical significance was defined as p <0.05.

† The cut-off values for age, BMI, SBP at baseline, number of antihypertensive drugs at baseline and compliance were chosen according to the mean values (see table 1, baseline characteristics).

Abbreviations: BMI: body mass index; CI: confidence interval; DBP: diastolic blood pressure; SBP: systolic blood pressure; TTR: time in target range.
